# A QSAR Study of Environmental Estrogens Based on a Novel Variable Selection Method

**DOI:** 10.3390/molecules17056126

**Published:** 2012-05-21

**Authors:** Zhongsheng Yi, Aiqian Zhang

**Affiliations:** 1 State Key Laboratory of Pollution Control and Resource Reuse, School of the Environment, Nanjing University, Nanjing 210093, China; 2 College of Chemistry and Bioengineering, Guilin University of Technology, Guilin 541004, China; 3 State Key Laboratory of Environmental Aquatic Chemistry, Research Center for Eco-Environmental Sciences, Chinese Academy of Sciences, Beijing 100038, China

**Keywords:** variable selection method based on variable interaction (VSMVI), QSAR, estrogen, logRBA, application domain

## Abstract

A large number of descriptors were employed to characterize the molecular structure of 53 natural, synthetic, and environmental chemicals which are suspected of disrupting endocrine functions by mimicking or antagonizing natural hormones and may thus pose a serious threat to the health of humans and wildlife. In this work, a robust quantitative structure-activity relationship (QSAR) model with a novel variable selection method has been proposed for the effective estrogens. The variable selection method is based on variable interaction (VSMVI) with leave-multiple-out cross validation (LMOCV) to select the best subset. During variable selection, model construction and assessment, the Organization for Economic Co-operation and Development (OECD) principles for regulation of QSAR acceptability were fully considered, such as using an unambiguous multiple-linear regression (MLR) algorithm to build the model, using several validation methods to assessment the performance of the model, giving the define of applicability domain and analyzing the outliers with the results of molecular docking. The performance of the QSAR model indicates that the VSMVI is an effective, feasible and practical tool for rapid screening of the best subset from large molecular descriptors.

## Abbreviations:

QSAR: quantitative structure-activity relationship
LOO: leave-one-out cross validation
VSMVI: variable selection method based on variable interaction
LMO: leave-multiple-out
CV: cross validation
LOOCV: leave-one-out cross validation
LMOCV: leave-multiple-out cross validation
MCCV: Monte Carlo cross validation
OECD: Organization for Economic Co-operation and Development
EDCs: endocrine disrupting chemicals
ER: estrogen receptor
MLR: multiple-linear regression
ASR: all-subsets regression
PLS: partial least squares
VSMP: variable selection and modeling method based on the prediction
EA: evolutionary algorithms
UFS: unsupervised forward selection
LASSO: least absolute shrinkage and selection operator
GA: genetic algorithms
*k*NN: *k*-nearest neighbor
RMSE: root-mean-square errors
PSO: particle swarm optimization
RMSEV: root-mean-square errors of leave-one-out cross validation
RMSEP: root-mean-square error of the test set
logRBA: middle logarithm of relative binding affinities
STD: standard deviation
RBA: relative binding affinities
VCCLAB: Virtual Computational Chemistry Laboratory
EDKB: endocrine disruptor knowledge base
CoMFA: comparative molecular field analysis
CoMSIA: comparative molecular similarity indices analysis
GMDH: group method of data handling
NCTR: National Center for Toxicological Research
CODESSA: comprehensive descriptors for structural and statistical analysis
HQSAR: hologram quantitative structure-activity relationship

## 1. Introduction

Estrogens play an important role in the growth, development, sustenance of a wide range of tissues, and especially in the physiology of the female reproductive system, the maintenance of bone density, and cardiovascular health [1]. It has been known that estrogen can increase the risk of cancer within tissues, particularly the female breast. Xenoestrogens have been found to mimic estrogen by binding to the ER as either an agonist or antagonist [2,3,4]. These xenoestrogens together with other xenobiotics (such as xenoandrogens, xenoprogesterone) are termed endocrine disrupting chemicals (EDCs). They become an emerging field and attract more and more attention from scientists and political institutions. There are a number of exogenous substances which can not only effect the function of the endocrine system but also produce influence on the homeostasis of all the process controlled by this system in humans and wildlife.

Generally, two prior ways can be used for screening of the EDCs from large-size chemicals. (1) High-throughput screening based on *in vivo* and *in vitro* tests. However, such tests are laborious, time-consuming, and expensive. It is impractical to carry out through toxicological tests on the large-size potential disrupting chemicals. Therefore, the number of experimental data available to characterize the endocrine disrupting effect of EDCs is very limited. (2) Prior to screen with quantitative structure-activity relationship (QSAR) techniques, which are among the successful strategies that can maximize the value of existing data, using them to predict unknown activities for existing or even not yet synthesized chemicals and to design safer alternatives that can substitute unsafe chemicals.

QSAR for predicting the binding affinity between ligand and hormone receptor have been proposed as screening tools to help prioritize untested compounds for more intensive investigations to assess potential effects on steroid signaling pathways [5]. Especially in screening of large numbers of chemicals and addressing the ability of xenobiotics to disrupt endocrine functions, it is impractical to experimentally test all chemicals that may possess endocrine disrupting activity. In this context, QSAR is an excellent tool to overcome these limitations. QSAR models have proven their utility, from both the pharmaceutical and toxicological perspectives, and play an essential role in toxicology as a priority setting tool for risk assessment. There are several comprehensive reviews of QSARs for xenoestrogens [6,7,8]. These QSAR models provide different perspectives on the interactions between the estrogen receptor and its ligands. For example, 3D-QSAR, such as comparative molecular field analysis (CoMFA) and comparative molecular similarity indices analysis (CoMSIA), mainly considers the interaction of xenoestrogens with estrogen receptor (ER) as a 3D process, and explain their biological activities primarily through the energy information of steric and electrostatic, but sometimes through hydrophobic or hydrogen bonding potential fields around of a set of aligned molecules. Although 3D-QSAR has been used successfully in many studies, its models are not straightforward because each molecule can exist in multiple conformations with different levels of stability and occurrence. 2D-QSAR interprets the mechanism of xenoestrogens binding to ER using physicochemical, topological and quantum-chemical descriptors, without receptors’ information, and it is relatively easy to set up a procedure to make them reproducible. In fact, 2D-QSAR and 3D-QSAR have their own advantages and disadvantages and cannot replace each other in QSAR study of EDCs screening.

The basic idea behind QSARs on estrogens is to find mathematical relationships between descriptors that reflect the structure and physicochemical properties of molecules and their relative binding affinities (RBA). With the rapid development of structural characterization techniques, it is no longer a difficult task to yield structure descriptors. For example, one can easily obtain 29 categories of descriptors using Dragon 6, and the total amount is 4,886 kinds. Subsequently, variable selection becomes more and more important to establish predictive and robust QSAR models according to Ockham’s razor principle [9,10]. Normally, variable selection represents a discrete optimization problem. Therefore, descriptor screening is rather complicated due to the 2*^m^*-1 possible combination of descriptors for a given data set comprising *m* descriptors, which may turn into real dimensional disaster facing a large variable candidate pool. Several techniques for optimizing variable selection have been reported, and two excellent review papers on variable selection have been published by González *et al.* [11] and Tsygankova [12]. In general, all variable selection techniques can be classified into two groups: (1) systematic search methods, which are common based on an all-subsets regression (ASR) [13,14,15] approach; examples are variable selection and modeling method based on the prediction (VSMP) [16], unsupervised forward selection (UFS) [17], and least absolute shrinkage and selection operator (LASSO) [18]. (2) Stochastic search methods, which include *k*-nearest neighbor (*k*NN) with simulated annealing [19], evolutionary or genetic algorithms (EA or GA) [20], particle swarm optimization (PSO) [21], and the ant colony optimization algorithm [22]. However, the systematic and stochastic search methods have their own limitations. Generally, the stochastic methods may not ensure that the same global optimal subset is found, while it is impossible to use systematic search scenarios for subset selection when there is a large variable candidate database.

On the other hand, the correlation coefficient (*q*^2^) or the root-mean-square errors of leave-one-out cross validation (RMSEV) are employed to assess the quality of QSAR model, especially during variable selection. However, there are some particular problems [23,24,25], such as over-fitting, under-estimation of the true predictive error, because leave-one-out cross validation (LOOCV) is an asymptotically inconsistent method [26,27,28]. Fortunately, the deficiencies of LOOCV can be overcome by using leave-multiple-out cross validation (LMOCV) [27,29], in which the data set described by *n* samples (or compounds) and *m* descriptors is split into two parts, and the first part (construction/ training set) containing *n_c_* samples is used for fitting a model (model construction), whereas the second part (validation/test set) including *n_v_* (*n_v_*= *n* − *n_c_*) samples is reserved to assess the predictive ability of the model (model validation). Clearly, if *n_v_* = 1, then LMOCV is LOOCV. In the procedure of cross-validation, LMOCV is repeatedly performed for a sufficiently large number of times (*N*).

The main aim of the present study is to obtain a good linear regression equation for predicting the estrogen binding to the estrogen receptor from a large size descriptor pool calculated from E-Dragon. A novel variable selection method based on variable interaction (VSMVI) with LMOCV is developed to select the optimal subset.

## 2. Results and Discussion

### 2.1. Construction and Validation of Model

**Figure 1:**
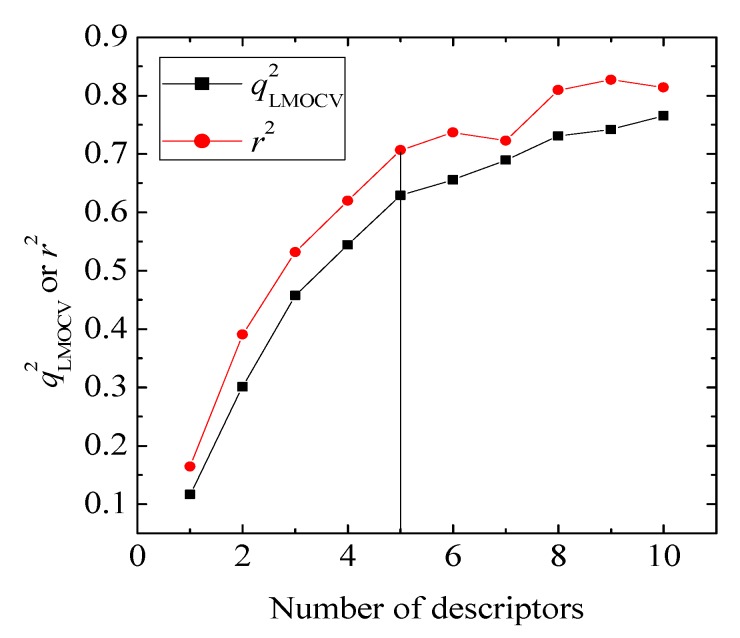
Correlation coefficients of LMOCV (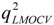) *vs.* number of descriptors.

**Figure 2:**
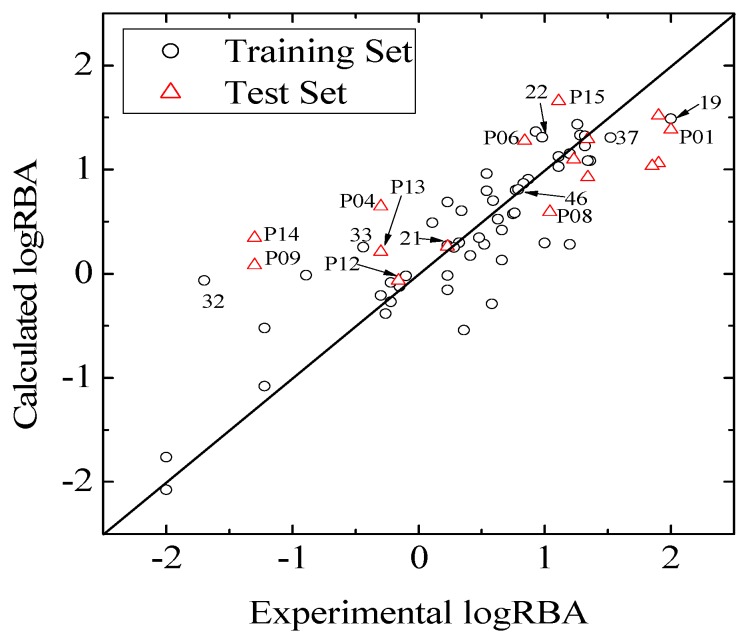
Plots of experimental logRBA *versus* calculated values of training and test sets.

The 760 descriptors of 53 compounds were used as the independent variable (*x*) and logRBA as the dependent variable (*y*) to establish the optimal model. Two-fold MCCV was employed to perform the LMOCV. Plots of 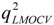 values against the number of descriptors (Figure 1), which provide guidance in deciding the number of descriptors for constructing models, suggest that the optimal models include five descriptors, because the increase in 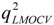 with the five descriptor is less than 5%. The names, descriptions, and types of the descriptors are listed in Table 1. As shown in Table S3 (supporting information), there is non-correlation between any two variances (correlation coefficient *r *< 0.8). The experimental and predicted logRBA values for the 53 compounds were summarized in Table S1 (supporting information) and the plots of the experimental logRBA values versus calculated values of the training and test sets were described in Figure 2. The best multiple linear regression model developed using the optimal subset was presented as Equation (1) and Table 2:


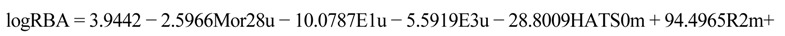
(1)

In *y*-randomization validation, the 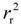 value of QSAR model [Equation (1)] is 0.0967 and the ^c^*r*^2^_p_ value 0.7040. Thus, it can be inferred that the QSAR model developed in the present study is not only the outcome of chance.

**Table 1:** Names and description of the descriptors in optimal models.

Name	Description	Descriptor Type
Mor28u	signal 28 / unweighted	3D MoRSE descriptors
E1u	1st component accessibility directional WHIM index / unweighted	WHIM descriptors
E3u	3rd component accessibility directional WHIM index / unweighted	WHIM descriptors
HATS0m	leverage weighted autocorrelation of lag 0 / weighted by mass	GETAWAY descriptors
R2m+	R maximal autocorrelation of lag 2 / weighted by mass	GETAWAY descriptors

**Table 2:** Some statistic parameters of the model.

	*n*	*m*	*r*^2^ or 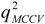	*RMSE*	*F*	*R*_p_
Estimation	53	5	0.7540	0.4275	28.8090	0.7043
MCCV	53	5	0.6375	0.5166		
LOOCV	53	5	0.6909	0.4792		
External test	16	5	0.5308	0.7098		

Consequently, to ascertain the predictive power of the QSAR model, validation of an external set may be more important. Hence, a predicted set containing 16 chemicals, which has been also used by Tong [30], was employed to validate the model. Statistical results of external validation for the model are as follows: the predictive correlation coefficient 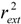 and root-mean-square error of the test set (*RMSEP*) are 0.5308 and 0.7098, respectively, which demonstrate that the derived model exhibits quite good predictive ability. The experimental and predicted LogRBA of 16 compounds in the test set are shown in Table S1 and Figure 2. 

### 2.2. Application Domain and Outlier Analysis of Models

**Figure 3:**
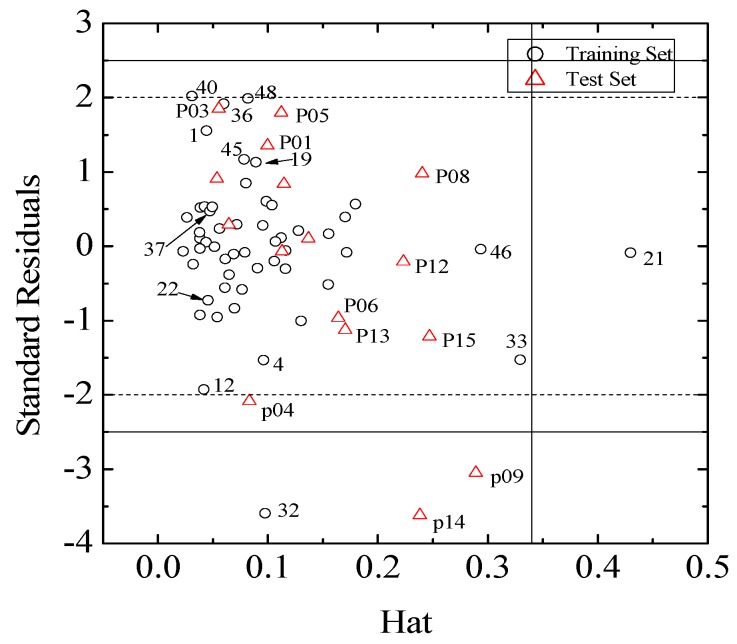
Williams plot for the training and test sets.

According to the OECD (Organization for Economic Co-operation and Development) principles for regulation of QSAR models, the domain of application must be defined and only the predictions for the xenoestrogens that fall in this domain may be considered reliable. The plot of standard residuals *versus* leverage values (the Williams plot), which can be used to obtain immediate and simple graphical detection of both logRBA outliers (*Y* outliers) and structurally influential compounds in a model (*X* outliers), was shown in Figure 3. 

The Williams plots (Figure 3) reveal that there is only one *X* outlier (the leverage value h* = 3 × (5 + 1)/53 = 0.34), compound **21** in the training set, and compounds **33** and **46** are very close to the leverage value. Commonly, the further away an *X* outlier is from the leverage, the greater of the impact of MLR regression coefficients is. Compared with the origin model, there is no significant difference from the regression coefficients of models when excluding compounds **33** and **46** (Table S4), due to the hat value (0.4374) being very close to the leverage value. On the contrary, the hat value of E1u and R2m+ in the model when excluding **21** increases remarkably. Actually, the structural differences between compound **21** and other compounds with the same skeleton (such as compounds **22**–**24**) are rather small. Nevertheless, considering the induced fit conformational change of key residue of estrogen receptor during ligand-receptor interaction, such is understandable. 

**Figure 4:**
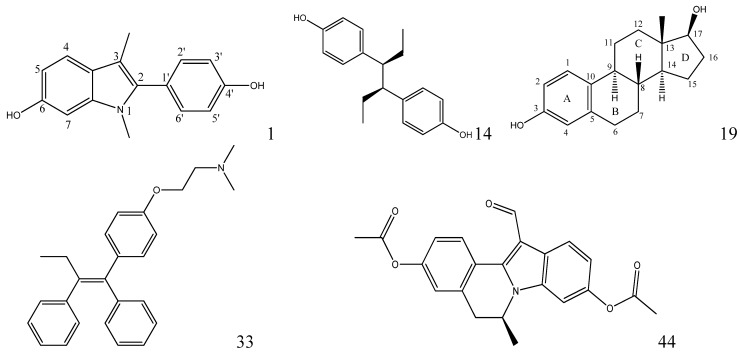
The chemical structures of various natural or synthetic estrogens used in this study.

**Figure 5:**
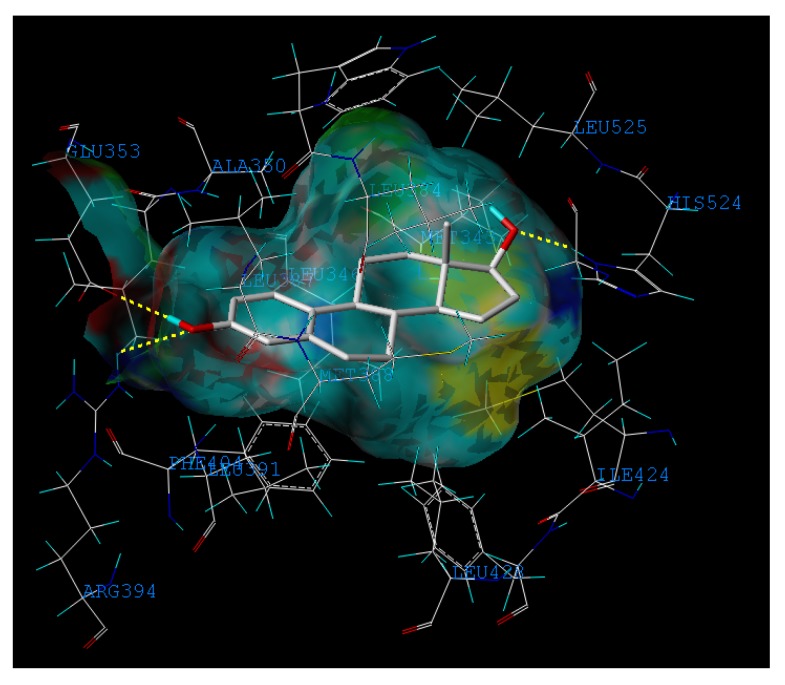
Representation of the binding mode of the **E2** into the crystal structure of ERα (PDB code: 1ERE)

**Figure 6:**
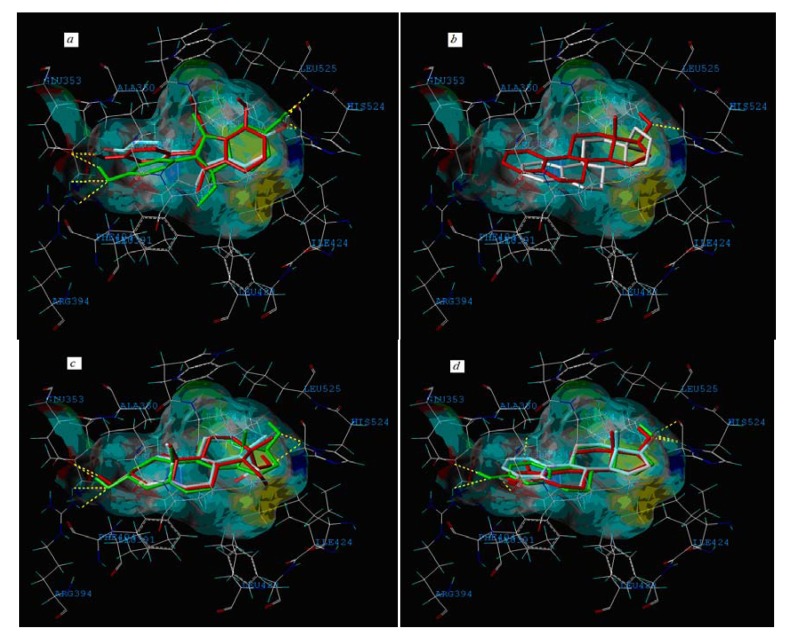
The interacting mode of compounds with ERα (PDB code: 1ERE). (**a**) The red is compound **37**, the green is compound **22** and the remaining one is compound **32**; (**b**) The red is compound **P08**, and the other is **P09**; (**c**) The green is **P12**, the red is **P13** and the remaining one is **P14**; (**d**) The green is **P01**, the red is **P04** and the remaining one is **P06**.

Moreover, compound **32** in the training set and **P09** and **P14** in the external test set are *Y* outliers respectively, since those compounds have standardized residuals greater than 2.5 standard deviation units. The prediction error of these three compounds is significantly larger than that of the other compounds. Specifically, the compound **32**, which is a *Y* outlier, is a 2-phenylindole with a 7-OH (for the 2-phenylindole backbone see Figure 4 and Table S2), whereas the hydroxyl group of other 2-phenylindoles locates at the 5- or 6-position for compounds **22** and **37**, individually. This is quite similiar to the situation of compound **21**, and the values of descriptors of compounds **32**, **22** and **37** are also very close. However, the logRBA values of the three compounds are quite different. Tong *et al*. [30] also obtained a similar result using CODESSA descriptors. Molecular docking analysis was adopted to get insight into the problem. The ERα-**E2** crystal structure shows that the 3-OH of **E2** establishes H-bonding interactions with Glu 353, Arg 394, and a water molecule, while the 17β-OH only forms one H-bond with His 524 (Figure 5, water molecule was not shown. Here, all compounds were docked into the crystal structure of ERá using SYBYL-X1.1). Clearly, a 3-OH is more important than the 17β-OH in ER binding [31,32]. Like the 17β-OH of **E2**, the 6-OH and 5-OH of the 2-phenylindoles play a similar role in complex stability and establish H-bonding interaction with His 524 for compounds **37** and **22**, and the distances between 6-OH or 5-OH and His 524 are 2.81 and 3.10 Å, respectively (Figure 6a). In addition, the 7-OH in compound **32** can hardly interact with the His 524 in rigid docking strategy since the distances between 7-OH and His 524 is 4.41Å. Necessary pocket plasticity should be introduced due to the low ligand flexibility of the compounds. Meanwhile, Fang *et al.* [33] have denoted that the orientation of the 3- and 17β-OH group of steroids as well as the distance (*d*_O–O_) between them govern binding affinity. If the two hydroxyl groups in a compund resemble the orientation and location as those of **E2** (*d*_O–O_ = 11.0Å), high estrogenic activity can be expected. Those with hydroxyl groups mimicing those of **17α-E2** (*d*_O-O_ = 10.4Å) may provide a low estrogenic activity. For compounds **22**, **32** and **37**, the distances (*d*_O–O_) are 11.41, 9.34 and 11.28Å, respectively, which explains the fact that logRBA values of compound **22** and **37** are greater than that of compound **32**.

The external test compound **P09** (logRBA < −1.30), which is a *Y* outlier and belongs to the steroids class, has a similar molecular structure to **P08** (logRBA = 1.04). There is no hydroxyl group on a ring of both compounds, whereas a 17β-OH (which could interact with His 524) occurs in compound **P08** but not in **P09** (Figure 6b). Similarly, the structural diversity for compounds **P12**, **P13** and **P14**, **P01**, and **P06** are also quite low, but their logRBA values are different (Figure 6c and Figure 6d).

### 2.3. Interpretation of the Built Model

In Equation (1), five descriptors in the final model were listed in Table 1. Mor28u is among the 3D-Molecule Representation of Structures based on Electron diffraction (3D-MoRSE) descriptors, which are based on the idea of obtaining information from the 3D atomic coordinates by the transform used in electron diffraction studies for preparing theoretical scattering curves, reflecting the molecular framework and structures of substituents. E1u and E3u as Weighted Holistic Invariant Molecular (WHIM) descriptors are geometrical descriptors based on statistical indices which are calculated on the projections of the atoms along principal axes. HATS0m and R2m+ are GEometry, Topology, and Atom-Weights AssemblY (GETAWAY) descriptors, which are chemical structure descriptors derived from the Molecular Influence Matrix. From the definition of 3D-MoRSE, WHIM and GETAWAY descriptors, these descriptors reflect the molecular character of 3D structure, size, branch and sharp. It indicates that the steric effect of the studied compounds may play an important role in creating H-bonds with the amino acids and water molecules in the ER binding pocket. The detail information about 3D-MoRSE, WHIM and GETAWAY descriptors has been discussed by Todeschini *et al*. [34,35]. In term of the standardized coefficient of descriptors (standard regression model, logRBA = −0.6332Mor28u − 0.5152E1u − 0.3684E3u − 0.5799HATS0m + 0.7946R2m+), the most significant descriptor is R2m+. The positive coefficient indicates that the larger the R2m+ value of a compound is, the higher the activity of the compound may be. Therefore, the molecular character of 3D structure, size, branch and sharp plays an important role for estrogenic activity of EDCs. The result is consistent with the results of Fang *et al.* [33].

### 2.4. Comparison with Other Models

Four models of selecting dataset developed by various authors were displayed in Table 3. Tong *et al*. [30,36] have developed three models using CoMFA, CODESSA and HQSAR descriptors, and PLS as the modeling method, respectively. The final models were used to predict the 16 compounds in the external test set. The performance of CoMFA model constructed by nine principal component, is the best of three methods with *r*^2^ = 0.97, 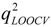 = 0.61 and *r*^2^_ext_ = 0.6718 (calculated by ourselves). However, the deficiency of large *r*^2^- 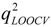 gap (0.97–0.61), which is often observed for CoMFA and similar models, may indicate the lower stability of this model. Compared to classical QSAR, the CoMFA method has obtained great success through employing the biological environment surrounding the molecules to interpret the mechanisms of action. However, there also present drawbacks for CoMFA, the main of which is that the complexity of the models is increased as a result of the requirement of 3D conformations, a suitable alignment rule of compounds and a large number of variables. It can make the method more difficult to reproduce a model or at least to apply it to new compounds if the alignment rules are too specific or not suitable for other chemical classes, and the range of chemicals that can be analyzed is limited. A PLS-HQSAR model, based on the use of fragment descriptors, was also derived from the same set of molecules. But the modeling performances are reduced compared to the CoMFA model (*r*^2^ = 0.93, 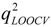 = 0.53), and a large gap between *r*^2^ and 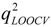 can be observed. The third model was constructed using 365 CODESSA descriptors and the best PLS model computed with these descriptors, including three components (*r*^2^ = 0.68, 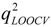 = 0.54). Afterward, Asikainen *et al.* [37] constructed a QSAR model for the same complete data set using the *k*NN method and 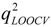 for the whole data set was 0.75. The performance of this QSAR model seems the better one. However, a large amount of descriptors (176) were used as model input, the statistic parameters of model were average result, and then the built model was found to be very difficult to interpret.

**Table 3:** Comparison with various models.

Model	Method	Descriptors	*r*^2^	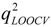	*r*^2^_ext_
Tong [30]	PLS	CoMFA	0.97	0.61	0.6712
Tong [30]	PLS	CODESSA	0.68	0.54	0.0217
Tong [36]	PLS	HQSAR	0.93	0.53	-
Asikainen [37]	KNN-QSAR	Dragon	0.86	0.73	-
This paper	MLR	Dragon	0.7540	0.6909	0.5308

In the present study, the five-descriptor model has a 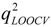 of 0.6909 and *r*^2^ of 0.7540 based on a simple and unambiguous MLR method. Although the value of *r*^2^ is less than those of CoMFA and HQSAR models and the *r*^2^_ext_ value is less than that of CoMFA, the value of 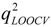 is greater than that of CoMFA and HQSAR methods and a small *r*^2^- 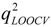 gap. In addition, our model has a explicit functional form, which makes it easy to use for other researcher. The comprehensive assessment (LOO and LMO cross validation, external validation and *y*-randomization test) provides satisfactory results. Therefore, our model may have certain advantages according to the above-mentioned discussion.

## 3. Experimental

### 3.1. Data Set

The logarithm of relative binding affinities (logRBA, which were calculated from a calf uterine estrogen receptor (calf ER) competitive binding assay with [^3^H] 17β-estradiol (E2).) of 53 natural, synthetic, and environmental chemicals for the estrogen receptor was obtained from the National Center for Toxicological Research (NCTR) endocrine disruptor knowledge base (EDKB, http://edka.fda.gov/databasedoor.html, accessed on January 2011). There have been QSAR models develped for this dataset by using CoMFA [30], COmprehensive DEcriptors for Structural and Statistical Analysis (CODESSA) [30] and Hologram Quantitative Structure-Activity Relationship (HQSAR) [36] methods. The corresponding logRBA value, and the EDKB ID number of the studied molecules are given in Table S1 (see Supporting Information). These compounds include several different chemical categories, such as 2-phenylindoles, steroidal estrogens, tamoxifen, hexestrol and isoquinolines (Figure 4). The external test set comprises the 16 estrogenic compounds obtained from literature [30]. The 2D-structures of all compounds are listed in Table S2 (Supporting Information).

### 3.2. Descriptor Generation and Preprocessing

The descriptors were calculated by E-Dragon, an online version of Dragon, which is available on the Virtual Computational Chemistry Laboratory (VCCLAB) [38,39]. A total of 1,666 molecular descriptors spanned 20 categories: Constitutional descriptors, Walk and path counts, Information indices, Edge adjacency indices, Topological charge indicates, Randic molecular profiles, RDF descriptors, WHIM descriptors, Functional group counts, Charge descriptors, Topological descriptors, Connectivity indices, 2D autocorrelation, Burden eigenvalues, Eigenvalue based indices, Geometrical descriptors, 3D MoRSE descriptors, GETAWAY descriptors, Atom-centered fragments, and Molecular properties. The definitions of these descriptors have been reviewed by Todeschini *et al.* [34,35].

Due to the different calculation theories used, the 1,666 molecular descriptors include extreme redundancy. To reduce the amount of calculation, a preselection of descriptors was implemented as follows: (1) the descriptors with standard deviations less than 0.00001 were excluded. (2) If the absolute values of correlation coefficient (|*R*|) between two descriptors were equal to and greater than 0.95 (|*R*| ≥ 0.95), either of them was deleted. The deleted descriptors take no part in the variable selection process, but they can be recovered at any time. (3) The descriptors with 90% zero values in *n* samples were excluded. Therefore, the descriptors were reduced to 760.

### 2.3. Variable Selection Method Based on Variable Interaction (VSMVI)

A single variable slightly correlated with a response may add useful information to QSAR model when taken in combination, due to variable interaction. An *n*-variable optimal subset should be obtained by combining an (*n *− 1)-variable optimal subsets with one other variable. Therefore, if we could determine a certain number of (*n *− 1)-variable optimal subsets, the *n*-variable optimal subsets could be obtained from these (*n *− 1)-variable optimal subsets combined with one other variable. This idea is the basis of VSMVI. Like VSMP [40], two statistic parameters were introduced in VSMVI. One was for the correlation coefficient between the variables, *r*_int_, and the other was for the correlation coefficient in LMOCV process, 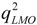. They were incorporated into the ASR procedure to accelerate the variable screening speed and control the quality of model, respectively. In fact, The VSMVI method has adopted the ideas of forward selection, VSMP and Group Method of Data Handling (GMDH) [41], and thus it has a high speed for screening variable. The two-order interaction is critically important, so the two-variable combination is the start of the screening. The VSMVI procedure includes two parts. One is the specified (*Ns*) optimal single variable and two variables subsets; these are selected using a similar VSMP technique and saved into an optimal subset pool. The other part is every subset of the last *Ns* optimal subset pool combined with one variable that is not in the selected subset, just in order to create a new subset. The new *Ns* optimal subsets are also saved into a new optimal subset pool. The speed of variable selection will be much quicker than VSMP, because the calculations necessary for variable combination are much fewer than those for the VSMP method. For example, for selecting a five-descriptor optimal subset from 53 descriptors, there are 2869685 combinations that need to be calculated. For VSMVI with 1,000 optimal subsets saved (*Ns *= 1,000), this drops to only 53,000 combinations.

**Figure 7:**
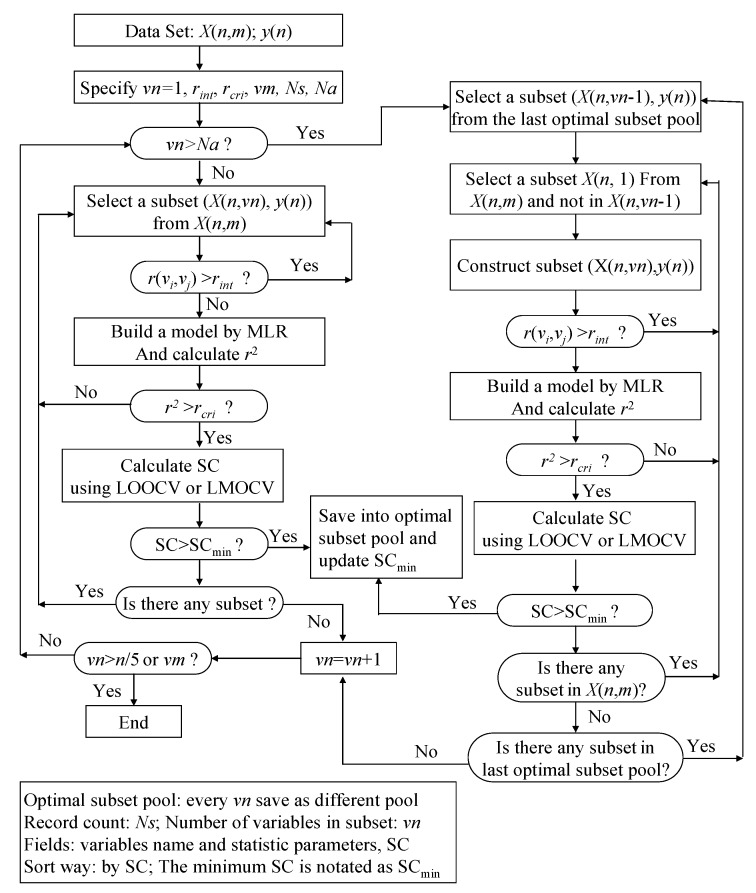
Variable selection method based on variables interaction.

The procedure of the VSMVI technique is applied to a *n*×*m* matrix *X *= (*x_ij_*), where *x_ij_* is the value of the *j*th variable for the *i*th compound, as shown in Figure 7. The detailed selection steps are described as follows:

(1) The values of *vn*, *r_int_*, *r_cri_*, *vm*, *Ns* and *Na* are specified, where *vn* is the number of variables in the current subset. In this paper, the *vn* is set as 1, 2, 3…, and 15. In other words, the largest number of variables for an optimal subset, *vm*, is designated as 15, but it should equal an integer *n*/5 if *vn* is larger than the integer of n/5. The *r_int_*, the allowable maximal threshold of the correlation coefficient between various pairs of the independent variables, is set to 0.9, but some authors set this to 0.75 [40]. The *r_cri_*, a control parameter used to determine whether the next cross validation step is performed or not, is increased with the number of variables and the initial value equals 0.1. The *Ns*, the number of subsets in the optimal subset pool, is set to 1000. Whether or not the second part of VSMVI is performed for selecting variables, the *Na* user-defined threshold is usually set to three.

(2) If the given *vn* is not greater than *Na*, a similar VSMP technique is used to select the optimal subset. The steps are as follows:

(a) A subset, *X*(*n*,*vn*), is systematically selected from the initial data set, *X*(*n*,*m*). All correlation coefficients, *r*(*v_i_*,*v_j_*), between all pairs of variables in the subset are calculated. If the value of any a *r*(*v_i_*,*v_j_*) is larger than the *r_int_* specified above, then the selection of the next subset is initiated. If *r_int_* remains larger than *r*(*v_i_*,*v_j_*), variable screening becomes quicker, because the cross validation procedure, which consumes the most time in variable selection, is avoided.
(b) If all values of all *r*(*v_i_*,*v_j_*)s are smaller than the *r_int_*, a multiple linear regression (MLR) model is built between the independent variable subset, *X*(*n*,*vn*), and the dependent variable set, *y*(*n*), and the correlation coefficient *r*^2^ of the model is calculated. If the value of *r*^2^ is smaller than the *r_cri_*, the next subset is selected continuously according to step (a).
(c) If the value of *r*^2^ is larger than *r_cri_*, a stop criterion (SC) is calculated. The SC can be *q*^2^, RMSEV (root-mean-square errors of validation) and so on, and in this paper, the correlation coefficient in LMOCV process, 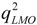 is used. If the value of SC is larger than SC_min_ (minimum SC in the optimal subset pool), the subset is plunged into the optimal subset pool. At the same time, the subset having the smallest value of SC (SC_min_) is replaced, and the SC_min_ is updated.
(d) If any subset still exists that has not been selected in the whole subset space, the process will return to step (a) to continue the selection of the next subset; otherwise *vn* is increased by one. If *vn* is not greater than *n*/5 or *vm*, the process will return to step (2), where *vm* is the maximum value of variables in the optimal subset.

(3) If *vn* is greater than *Na*, the second part of VSMVI is performed. The steps are as follows:

(a) A subset, *X*(*n*,*vn* − 1), is systematically selected from the last optimal subset pool, in which the number of variables is *vn* − 1.
(b) A subset, *X*(*n*,1), is also systematically selected from the initial data set, *X*(*n*,*m*), and the variable in *X*(*n*,1) is not included in the selected *X*(*n*,*vn *− 1). A new subset, *X*(*n*,*vn*), is constructed from *X*(*n*,1) and *X*(*n*,*vn*-1). All correlation coefficients, *r*(*v_i_*,*v_j_*), between all pairs of the variables in the subset are calculated.
(c) If the value of any *r*(*v_i_*,*v_j_*) is larger than the *r_int_* specified above, then the process returns to (b) to continue the selection of the next subset. If all values of all *r*(*v_i_*,*v_j_*)s are smaller than the *r_int_*, a MLR model is built between the independent variable subset, *X*(*n*,*vn*), and the dependent variable set, *y*(*n*), and the correlation coefficient *r*^2^ of model is calculated. If the value of *r*^2^ is smaller than the *r_cri_*, the next subset is selected continuously according to step (b).
(d) If the value of *r*^2^ is larger than *r_cri_*, the SC is calculated. If the value of SC is larger than SC_min_, the subset is plunged into optimal subset pool and the subset that has the smallest value of SC (SC_min_) is replaced. At the same time, the SC_min_ is updated. If any subset still exists that has not been selected in the *X*(*n*,*m*), the process will return to step (b) to continue the selection of the next subset, or will go to step (e).
(e) If any subset still exists that was not selected in the last optimal subset pool, the process will return to step (b) to continue the selection of the next subset, or will go to step (a); otherwise *vn* will be increased by one. If *vn* is not greater than *n*/5 or *vm*, the process will return to step (3) or the optimal process will be ended.

### 3.4. Leave-Multiple-Out Cross Validation

Commonly, the function of a QSAR model based on MLR is related to the correlation coefficient(*r*^2^) and root-mean-square errors (RMSE). However, excellent value of *r*^2^ or RMSE is insufficient indicators of model validity. Thus, cross validation (CV), or more accurately, LOOCV and LMOCV have been developed for assessing model quality [42]. The correlation coefficient (*q*^2^) or the RMSEV are employed as objective functions, especially during variable selection.

In cross validation, the dataset described by *n* compounds and *m* descriptors is split into two parts. LMOCV is repeatedly performed for a sufficiently large number of times (*N*). Meanwhile, the effect of LMOCV is twofold: (1) It is more difficult to fit a good model with fewer construction datasets, where it is only of size *n*/2, with *n* being the number of compounds in the construction dataset. (2) The model is assessed with a larger validation dataset. Hence, the construction and the validation set are less similar in each split than in LOOCV. This allows for better estimation of the predictive ability of the model. Both mechanisms prevent LMOCV from learning the idiosyncrasies of the dataset, and as a result, the degree of overfitting in variable subset regression is reduced [27,43,44]. Thus, in the present study, the VSMVI was employed to select the optimal subset with the aid of twofold LMOCV which was performed by Monte Carlo cross validation (MCCV) [45], and the MLR method was used to construct the QSAR model. All computations were performed by an in-house program called general variable selection and modeling program.

For the multiple-linear model, the correlation coefficient *r*^2^ or the *RMSE* of estimation are used to assess model fitting capability. The external predicted capability is evaluated using the correlation coefficient 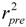 of test set (external set). They can be calculated by the following equations:


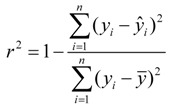
(2)


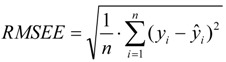
(3)


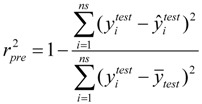
(4)

where *y_i_* and 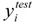 are the experimental values (logRBA in present study) of the *ith* compound in the training and test sets; 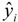 and 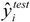 represent the estimators of the *ith* compound obtained via the linear model; 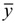 and 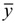
_*test*_ are corresponding average values; and *n* and *ns* denote the number of compounds of the training and test sets, respectively. 

The average of the correlation coefficient or the *RMSE* of LMOCV with *N* runs, 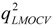 or *RMSEV^LMOCV^*, is employed to assess the average prediction error of a model. Corresponding standard deviation *STD*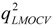 or *STDRMSEV^LMOCV^* is used to evaluate the robustness of a model. They are defined as follows:


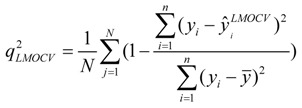
(5)


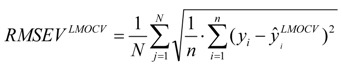
(6)


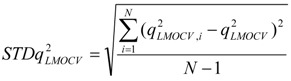
(7)


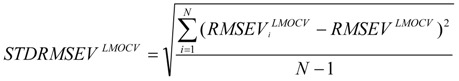
(8)

where 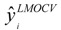 is the estimator of the dependent variable value obtained via *ith* iteration in LMOCV; *N* is the number of iterations; and 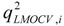 and 
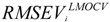
 are the correlation coefficient and *RMSEV* of *ith* iteration, respectively.

### 3.5. Application Domain

No matter how robust, significant, and validated a QSAR model may be, it cannot be expected to predict the modeled activity reliably for the entire universe of chemicals. Therefore, the domain of application must be defined and the predictions only for those chemicals that fall in this domain may be considered reliable [46,47,48]. However, the domain of model applicability is rarely given for QSAR investigations though it would be essential [49].

The measure of how far a chemical is from the domain of applicability of a model is its leverage values (Hat), *h_i_*, defined as: 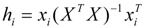 (I = 1, 2… *n*), where *x_i_* is the descriptor row vector of the query compound, and *X* is the *n* × (*k*-1) of *k* model descriptor values for *n* training set. Control leverage h* is generally fixed at 3*k*/*n*, where *k* is the number of model parameters (including the constant term of the MLR model), and *n* is the number of compounds used to construct the model. A leverage greater than control value h* means that the predicted logRBA is the result of substantial extrapolation of the model, and therefore may be unreliable [46]. The compounds with leverage greater than control value h* are identified as *X* outliers, which affect model performance, whereas those with standardized residuals greater than 2.5 standard deviation units are identified as *Y* outliers.

### 3.6. Chance Correlation Validation

The *y*-randomization validation is commonly used as the internal validation method [50]. It can check the overfitting and chance correlation between the dependent variable and the descriptors. In this internal validation method, if in each case the scrambled or randomized *y* data (dependent variable) give much lower *r*^2^ and *q*^2^ values than the original data (usually *q*^2^ of LOOCV/LMOCV is smaller than zero, and only in very few cases were the *q*^2^ values above zero), then one can feel confident about the relevance of the “real” QSAR model. In order to enhance the precision of the probability level, hundreds of runs of randomized data are usually required. In this study, one thousand runs of *y*-randomization validation were performed. Additionally, another parameter 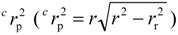
() [51] was also calculated to check the distance of QSAR models from chance models, where the 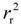 means the squared mean correlation coefficient of random models. 

## 4. Conclusions

In this paper, a five-descriptor QSAR model between the logRBA of compounds to the estrogen receptor and molecular descriptors of training sets was developed through applying 20 categories of molecular descriptors to characterize the molecular structures of 53 natural, synthetic, and environmental chemicals. A novel variable selection technique, VSMVI (variable selection method based on variable interaction) with twofold LMOCV was used to select the optimal subset. The results reveal that the models are significant, robust, and have satisfactory predictive ability. The 3D-MoRSE, WHIM and GETAWAY descriptors, which reflect the molecular character of 3D structure, size, branch and sharp, are the most important. The application domain reflects that the outliers are mainly within groups of chemicals with similar molecular structure and larger difference logRBA values. The molecular docking analyses further explain the fact that the different positions of the OH-group affect the ability of hydrogen bond formation between the ER receptor and the molecular with similar structure. Furthermore, during the development of the models, OECD principles for QSAR were fully considered. All results demonstrate that the proposed QSAR model is robust and satisfactory, and VSMVI is an effective method to select descriptors.

## Supplementary Materials

Supplementary materials can be accessed at: http://www.mdpi.com/1420-3049/17/5/6126/s1.
