# Supplementary material for: A QSAR Study of Environmental Estrogens Based on a Novel Variable Selection Method
Source: Molecules. 2012 May 21;17(5):6126–45. doi: 10.3390/molecules17056126 (PMC6268217; doi:10.3390/molecules17056126)
Supplement: Supplementary file 1 [file molecules-17-06126-s001.pdf]

Supplementary Materials

Table S1. Experimental, predicted log RBA and residual errors for the training set and the test set.

No.	EDKB-ID/Name	Experimental	Calculated
1	223	1.00	0.2947
2	239	1.36	1.0860
3	253	−2.00	−1.7647
4	254	−1.22	−0.5230
5	249	0.34	0.6050
6	247	0.23	−0.0196
7	235	1.20	1.1523
8	207	0.41	0.1738
9	204	0.11	0.4899
10	227	0.77	0.8015
11	236	0.54	0.9603
12	252	−0.89	−0.0142
13	210	−0.22	−0.2716
14	110	1.34	1.0832
15	221	0.93	1.3639
16	246	0.54	0.7945
17	250	0.87	0.9072
18	228	1.32	1.2263
19	87	2.00	1.4873
20	209	0.32	0.2966
21	240	0.23	0.2708
22	234	0.98	1.3101
23	211	0.28	0.2516
24	233	0.66	0.4189
25	243	−0.26	−0.3861
26	113	0.83	0.8673
27	230	−0.10	−0.0220
28	226	1.11	1.0270
29	242	0.23	−0.1553
30	99	0.75	0.5724
31	208	−0.15	−0.1218
32	241	−1.70	−0.0672
33	112	−0.44	0.2547
34	248	−0.22	−0.0865
35	229	1.28	1.3288
36	219	0.58	−0.2913
37	224	1.52	1.3052
38	251	−1.22	−1.0813
39	244	−2.00	−2.0755
40	220	1.20	0.2823
41	231	0.76	0.5844
42	206	−0.30	−0.2089
43	205	0.52	0.2796
44	202	0.23	0.6873

Table S1. *Cont.*

No.	EDKB-ID/Name	Experimental	Calculated
45	237	0.66	0.1298
46	91	0.79	0.8094
47	222	0.63	0.5218
48	238	0.36	−0.5434
49	245	0.48	0.3460
50	232	1.26	1.4348
51	203	0.59	0.7014
52	225	1.11	1.1233
53	310	1.32	1.3233
P01	17β-Estradiol	2.00	1.3830
P02	17α-Estradiol	1.34	0.9264
P03	16α-Estradiol	1.90	1.0602
P04	1-OH-Estradiol	−0.30	0.6460
P05	2-OH-Estradiol	1.85	1.0334
P06	4-OH-Estradiol	0.84	1.2777
P07	3-Hydroxyestratrien	1.90	1.5193
P08	Estratrien-17β-ol	1.04	0.5946
P09	Estratrien	−1.30	0.0840
P10	Estrone	1.34	1.2919
P11	Estriol	1.23	1.0969
P12	5-Androstene-3β,17β-diol	−0.16	−0.0667
P13	5α-Androstane-3β,17β-diol	−0.30	0.2107
P14	5α-Androstane-3a,17β-diol	−1.30	0.3439
P15	Moxestrol	1.11	1.6598
P16	Zindoxifen	0.23	0.2612

Table S2. 2D molecular structures of the training set and the test set.

1(1.00)	2(1.36)	3(−2.00)	4(−1.22)	5(0.34)	6(0.23)
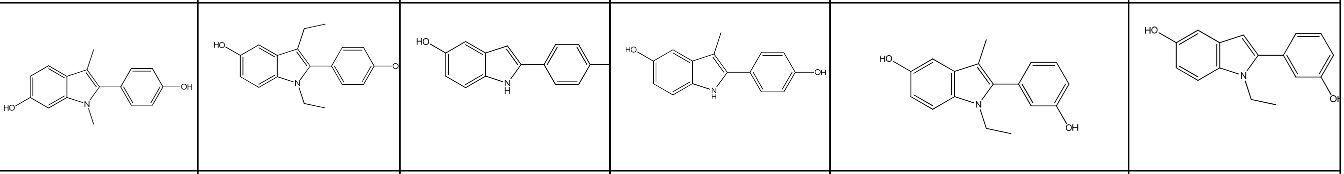					
7(1.20)	8(0.41)	9(0.11)	10(0.77)	11(0.54)	12(−0.89)
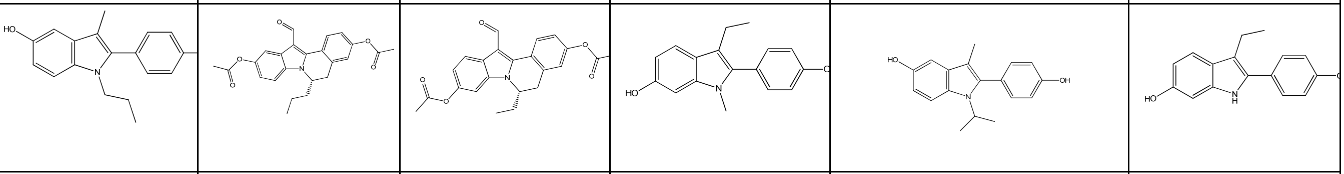					
13(−0.22)	14(1.34)-hexestrol	15(0.93)	16(0.54)	17(0.87)	18(1.32)
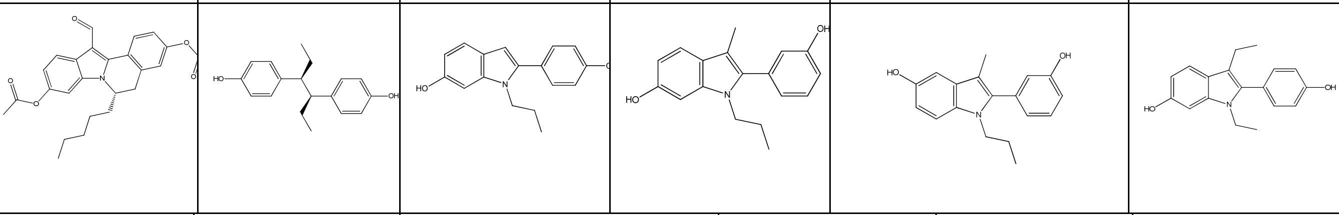					
19(2.00)	20(0.32)	21(0.23)	22(0.98)	23(0.28)	24(0.66)
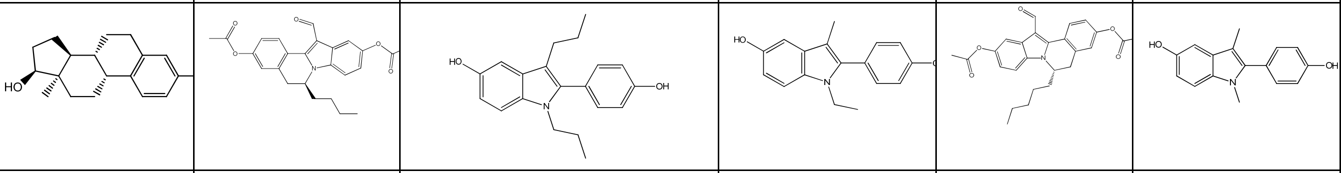					
25(−0.26)	26(0.83)	27(−0.10)	28(1.11)	29(0.23)	30(0.75)
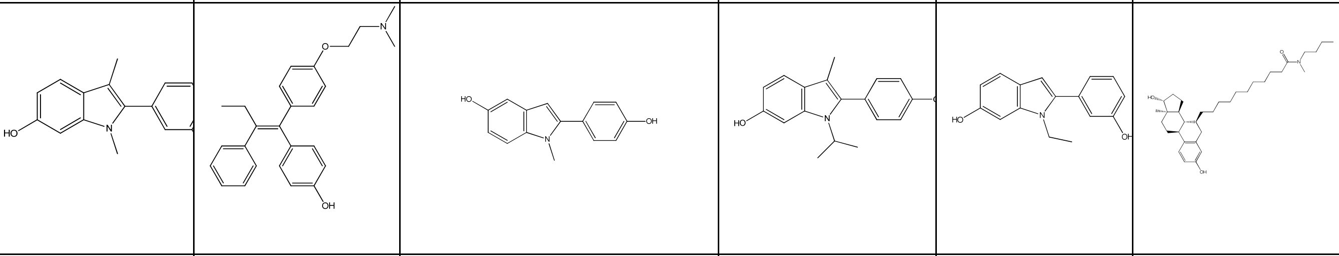					
31(−0.15)	32(−1.70)	33(−0.44)	34(−0.22)	35(1.28)	36(0.58)
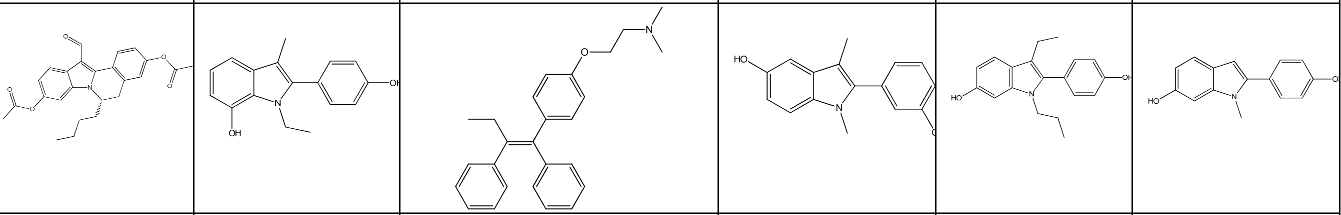					
37(1.52)	38(−1.22)	39(−2.00)	40(1.20)−7a	41(0.76)-18a	42(−0.30)-5b
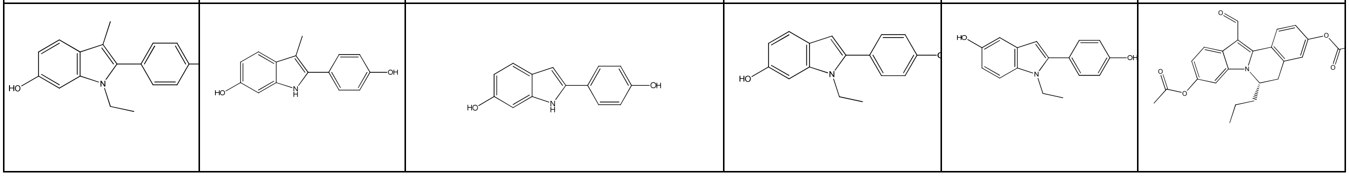					

Table S2. *Cont.*

43(0.52)	44(0.23)	45(0.66)	46(0.79)	47(0.63)	48(0.36)
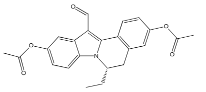	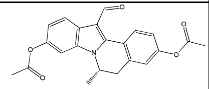	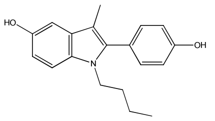	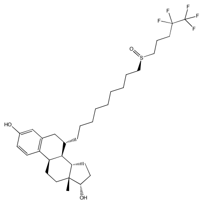	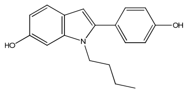	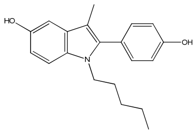
49(0.48)	50(1.26)	51(0.59)	52(1.11)	53(1.32)	
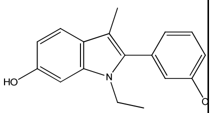	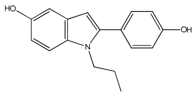	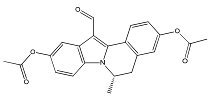	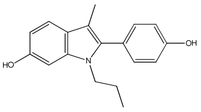	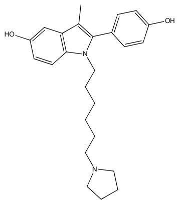	
P01(2.00)	P02(1.34)	P03(1.90)	P04(-0.3)	P05(1.85)	P06(0.84)
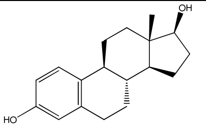	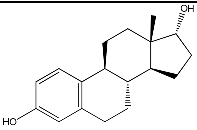	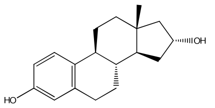	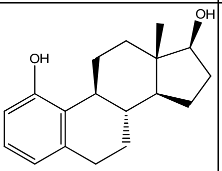	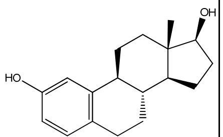	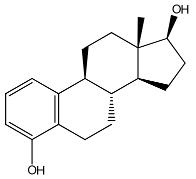
P07(1.90)	P08(1.04)	P09(-1.3)	P10(1.34)	P11(1.23)	P12(-1.6)
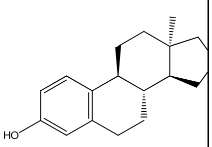	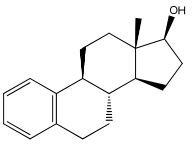	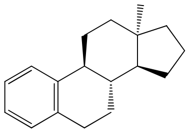	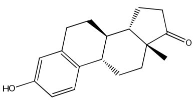	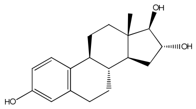	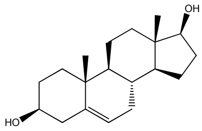
P13(-0.30)	P14(-1.30)	P15(1.11)	P16(0.23)		
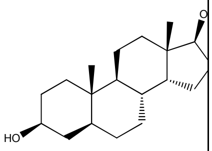	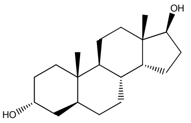	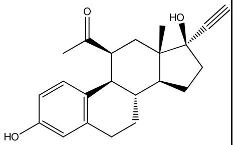	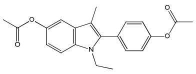		

Table S3. Self-correlation coefficient of the selected optimal subset.

	Mor28u	E1u	E3u	HATS0m	R2m+	logRBA
Mor28u	1.0000					
E1u	0.0645	1.0000				
E3u	−0.2899	0.2470	1.0000			
HATS0m	0.4121	0.2899	−0.3957	1.0000		
R2m+	0.3260	0.5435	−0.2544	0.7586	1.0000	
logRBA	−0.5395	−0.3833	−0.2848	−0.2417	−0.0381	1.0000

Table S4. Comparison of the regression coefficients excluded *X*-outlier with included one.

	**Constant**	**Mor28u**	**E1u**	**E3u**	**HATS0m**	**R2m+**
All	3.5885	−2.6520	−8.1220	−5.4385	−26.0613	78.2259
Exclude No. 1	3.5213	−2.6788	−8.5427	−5.0645	−25.3729	79.1847
Exclude No. 12	3.5654	−2.5862	−8.2232	−5.3914	−25.7159	79.2721
Exclude No. 25	3.6275	−2.6401	−8.3096	−5.3587	−26.4440	79.2488
Exclude No. 36	3.6600	−2.6905	−8.8949	−5.1451	−26.0292	81.1301
Exclude No. 43	3.5083	−2.6845	−8.2492	−5.2801	−25.5089	78.6424
Exclude No. 46	3.5834	−2.6429	−8.2602	−5.3534	−26.1470	79.1706
Exclude No. 33	***3.3083***	−2.8749	***−7.4240***	−4.8942	***−24.5714***	***70.7414***
Exclude No. 21	***3.9442***	−2.5966	***−10.0787***	−5.5919	***−28.8009***	***94.4965***
